# Microbial Load, Prevalence and Antibiograms of Salmonella and Shigella in Lettuce and Green Peppers

**DOI:** 10.4314/ejhs.v20i1.69431

**Published:** 2010-03

**Authors:** Biniam Guchi, Mogessie Ashenafi

**Affiliations:** 1 Department of Biology, Addis Ababa University, P.O. Box 1176, Addis Ababa, Ethiopia; 2 Institute of Pathobiology, Addis Ababa University, P.O. Box 1176, Addis Ababa, Ethiopia

**Keywords:** Lettuce, Green Pepper, microbial load, drug resistance

## Abstract

**Background:**

Human food borne infections traditionally are acquired through the ingestion of foods of animal origin. Fresh fruits and vegetables are major vehicles for the transmission of the food-borne infections. In Ethiopia, there is a tradition of consuming raw vegetables, particularly lettuce and green pepper, without adequate treatment. The objective of this study was to investigate the microbial load of fresh lettuce and green pepper, used as salad vegetables, and to assess the prevalence and antibiotic resistance of Salmonella and Shigella spp. isolated from lettuce and green pepper.

**Methods:**

A total of eighty samples of lettuce and green peppers were purchased from different outlets in Addis Ababa and analyzed for their load of various microbial groups and flora analysis was conducted following standard microbiological methods. The presence of Salmonella and Shigella and their antibiotic resistance was also determined.

**Results:**

Over 90% of the vegetable samples had aerobic mesophilic counts of ≥ log 6 cfu/g. Ninety seven percent of the lettuce and 58% of the green pepper samples had enterobacteraceae counts of ≥ log 5 cfu/g. Coliforms were encountered at counts ≥ log 4 cfu/g in 48% and 35% of lettuce and green pepper samples, respectively. Over 80% of vegetable samples harbored staphylococci with counts ranging from log 4 to log 6 cfu/g. More than 88% of lettuce and 18% of green pepper samples had yeast and mold counts ≥ log 4 cfu/g. The aerobic mesophilic flora of the vegetable samples was dominated by Bacillus and Micrococcus spp. Salmonella and Shigella were isolated from eight (10%) and 24 (30%) samples, respectively. All of the Salmonella and 97% of Shigella isolates showed resistance to penicillin. Ampicillin resistance was observed in 42% of Salmonella and 79% of Shigella isolates. Multiple drug resistance was seen in 8 and 24 isolates of Salmonella and Shigella isolates, respectively.

**Conclusion:**

The majority of lettuce and green pepper samples had high microbial load and multiple drug resistant pathogens were also isolated from some samples. As lettuce and green pepper, when used as salad vegetables, do not get any further heat treatment, thorough washing and considerably longer exposure of the vegetables to food grade chemicals is recommended to kill pathogens and significantly reduce the microbial load.

## Introduction

Shortly after some major human pathogens were recognized as being spread from animal reservoirs, fresh fruits and vegetables emerged as new vehicles for the transmission of diseases (1). Human infections traditionally are acquired via the ingestion of contaminated foods and drinks (2). This becomes an important issue when coupled with the trend of people consuming more vegetables and fruits for health and nutritional reasons. Irrigation with poor-quality water is a major source of contamination to fruits and vegetables with foodborne pathogens. Like wise, the use of raw animal manure for fertilizer can increase the threat of contamination of fruits and vegetables (3).

There are a number of reports indicating that raw vegetables may harbor potential foodborne pathogens (3–6). *Listeria monocytogenes, Salmonella* (5), and Escherichia coli (6) have been isolated from raw vegetables. Enteric pathogens have been found on a wide variety of produce including lettuce, tomatoes, and cantaloupes (3). Outbreaks of salmonellosis have been attributed to the consumption of contaminated tomatoes (7).

Currently, there are numerous local outlets that distribute fresh produce in Addis Ababa. The major sources of vegetables are farms in different parts of the city which use heavily contaminated water for irrigation. Various undesirable microorganisms may be present at the time of purchase in grocery stores. This becomes particularly important in salad vegetables which are prepared and consumed without heat treatment. Information on the microbial safety of food items in Ethiopia is limited. There are very few reports on the microbial quality and prevalence of foodborne pathogens found on fresh produce in Ethiopia (8, 9).

The objective of this study is to investigate the microbial load and antibiotic resistance of *Salmonella* and *Shigella* isolates from lettuce and green pepper.

## Materials and Methods

**Collections of samples:** A total of 80 fresh produce samples were purchased at different sampling days from different outlets in Addis Ababa, Ethiopia between November, 2007–April 2008. The samples consisted of 40 lettuce and 40 green pepper. All samples were collected aseptically and immediately brought to the Microbiology laboratory at Biology department, Addis Ababa University for analysis. Microbiological analysis was conducted within three hours of sample collection.

**Microbiological analyses:** For microbiological analyses, 25g of sample was aseptically removed from each sample using a sterile scalpel and vigorously shaken in 225ml of sterile 0.1% (w/v) bacteriological peptone water (Oxoid) for 3 minutes. Appropriate serial dilutions of the suspension were then spread-plated on a suitable agar medium.

Aerobic mesophilic bacteria were counted using Plate Count (PC) Agar (Oxoid) plates incubated at 30°C for 72 hours. For Enterobacteriaceae, Violet Red Bile Glucose Agar (Oxoid) was used and plates were incubated at 30°C for 24 hours. All purple colonies were counted as members of Enterobacteriaceae. Coliforms were counted on Violet Red Bile Agar (Oxoid) after incubating plates at 30°C for 24 hours. Red to pink colonies, surrounded by precipitated bile, were counted as coliforms. For staphylococci, Mannitol Salt Agar (Oxoid) was surface plated and incubated at 30°C for 36 hours. Bacterial spores were counted after heating the suspension for ten minutes in water bath (80°C) and spread-plating 0.1 ml of appropriate dilutions on the pre dried surface of PC plates. Colonies were counted after incubation at 30 to 32° C for 24 hours. Counts of yeasts were determined on Chloramphenicol Bromophenol Blue Agar plates incubated at 25–28°C for three to five days. Chloramphenicol-Bromophenol blue agar consisted of (g/l distilled water) yeasts extract (Oxoid) 5.0, glucose 20, choramphenicol 0.1, Bromophenol-blue 0.01, agar 15, pH 6.0–6.4. Smooth (non-hairy) colonies without extension at periphery (margin) were counted as yeasts. Microbial counts were transformed to log10 cfu/g.

For flora analysis about 10 to 15 colonies were picked randomly from countable PC plates and purified by repeated plating and characterized to the genus level using the following tests: cell morphology, KOH test (10), oxidation fermentation (O/F) test (11), catalase test and cytochrome oxidase test (12).

For isolation of *Salmonella* and *Shigella* spp. vegetable samples (25 g) were added to 225 ml buffered peptone water, vigorously shaken and the suspension incubated at 37°C for 24 hours for the metabolic recovery and proliferation of cells. From this, 1ml of culture was transferred into separate tubes each containing 10 ml of Selenite broth, Selenite Cystein Broth, Tetrathionate broth, Mannitol Selenite broth (all from Oxoid). Selenite Cystein and Mannitol Selenite broths were incubated at 37°C for 24 hours and Tetrathionate broth was incubated at 43°C for 48 hours in water bath. After secondary enrichment, culture from each enrichment broth was separately streaked on plates of MacConkey Agar, Salmonella-Shigella (SS) Agar and Xylose Lysine Desoxycholate (XLD) medium (all from Oxoid). Characteristic colonies from each selective medium were picked, purified and tested biochemically on Triple Sugar Iron Agar (Oxoid), Lysine Iron Agar (LIA) (Oxoid), Urea Agar (Oxoid), Simmons Citrate Agar (Oxoid) and SIM Medium (Oxoid). The ability of *Salmonella* to ferment mannitol, glucose or sucrose was assessed using a fermentation broth containing the corresponding sugars. Fermentation tubes contained inverted Durham tubes to detect gas production.

**Drug susceptibility testing:** Antimicrobial susceptibility of *Salmonella* and *Shigella* isolates was tested on Mueller-Hinton agar plates following the standardized disk diffusion technique (13) with Oxoid drug discs: Ampicillin (Amp), (10iu); Chloramphenicol (Chl), (30µg); Gentamycin (Gen), (10µg); Tetracycline (Tet), (30µg); Ciproflaxin (Cip), (5µg); Ceftriaxone (Cef), (30µg); Penicillin G (Pen), (10ug); Streptomycin (Str), (10µg); Kanamycin (Kan), (30µg); Polymaxin B (Pol),(100iu); Amoxicillin, (Amo), (2 µg).The reference strains, S. aureus (ATCC 6538) and E. coli (ATCC 25922), sensitive to all the drugs used in this study, were routinely tested. Interpretation of readings as sensitive, intermediate or resistant was made according to a chart. Intermediate readings were few and therefore considered as sensitive for the purpose of assessing the data.

### Data analysis

Descriptive statistics was used to compute percentage and mean based on n>30. To see if there was significant variation in counts within samples in each type of lettuce and green pepper, coefficient of variation (CV) was calculated. CV indicated inconsistency in the level of microbial count and 10% level indicated significant variation in counts.

## Results

Both vegetable types contained a variety of microbial groups. Aerobic mesophilic bacteria at counts were higher than log 7 cfu/g. Coliforms and enterobacteriaceae were found at levels higher than log 4 cfu/g. There was no significant variation among counts of aerobic mesophilic bacteria among the lettuce samples (CV<10%). Counts of other bacterial groups, however varied significantly (CV>10%) among samples of both vegetable types (Table 1).

**Table 1:** Microbial counts (log cfu/g) of lettuce and green pepper purchased from supermarkets in Addis Ababa

	Lettuce			Green pepper		
Bacterial groups	Mean	S.D.^1^	%CV^2^	Mean	S.D.	%CV
**Aerobic mesopiles**	**7.51**	**0.74**	**9.84**	**7.53**	**0.97**	**12.86**
**Coliforms**	**4.97**	**0.83**	**16.78**	**4.06**	**0.77**	**19.03**
**Enterobacteriaceae**	**5.08**	**0.59**	**11.75**	**4.84**	**0.88**	**18.19**
**Spore**	**3.50**	**0.63**	**17.88**	**3.47**	**0.79**	**22.88**
**Staphylococci**	**4.55**	**0.63**	**13.9**	**4.97**	**0.75**	**15.12**
**Yeast and molds**	4.51	0.84	18.70	4.05	0.69	17.27

1 S.D. Standard deviation

2 C.V., Coefficient of variation

The aerobic microflora of the vegetables was dominated by a variety of Gram positive and Gram negative bacterial groups. *Bacillus* and *Micrococcus* species consisted over 50% of the total flora. Among the Gram positive isolates green pepper samples yielded more of these than the lettuce samples. In contrary, l Gram negative isolates dominated more of the lettuce samples. *Pseudomonas* isolates were the dominant among Gram negative isolates (Table 2).

**Table 2:** Dominant bacteria in lettuce and green pepper purchased form supermarkets in Addis Ababa.

Sample type	No of isolates	Percent							
		Enterobacteriaceae	*Pseudomonas*	*Aeromonas*	*Acinetobacter*	*Bacillus*	*Micrococcus*	*Staphylococcus*	Other G+ rods
Lettuce	229	8	16	13	7	30	17	1	8
Green pepper	378	4	8	3	1	30	32	4	18
Total	607	6	12	8	4	30	24.5	2.5	13

About 50% of the vegetable isolates had aerobic mesophilic bacterial counts higher than log 6 cfu/g. Over 60 samples had coliform and enterobacteriaceae count between log 3 and log 6 cfu/g. The other microbial groups showed different distribution frequencies (Table 3).

**Table 3:** Frequency distribution of various microbial groups on lettuce (L) and green pepper (G)

Microbial groups	Sample type	Log (cfu/g)								
		<2	2–2.99	3–3.99	4–4.99	5–5.99	6–6.99	7–7.99	8–8.99	>9
AMB^1^	L	0	0	0	0	4	21	13	1	1
	G	0	0	0	0	4	12	12	11	1
Enterobacteriaceae	L	0	0	1	9	25	5	0	0	0
	G	0	6	11	17	5	1	0	0	0
Coliforms	L	0	2	4	15	15	4	0	0	0
	G	0	2	14	11	13	0	0	0	0
Aerobic spores	L	2	15	20	3	0	0	0	0	0
	G	1	11	15	13	0	0	0	0	0
Staphylococci	L	0	0	3	15	22	0	0	0	0
	G	0	0	5	24	8	3	0	0	0
Yeasts and molds	L	0	3	2	13	19	3	0	0	0
	G	0	15	18	6	1	0	0	0	0

1 AMB, aerobic mesophilic bacteria

Almost all *Salmonella* isolates were resistant to Pen and Amo. They also showed limited resistance to Str, Cep and Amp. Most of the *Shigella* isolates were also resistant to Pen, Amo, and Amp. Over a third of the isolates were resistant to Str, Tet and Cep (Table 4).

**Table 4:** Frequency of resistance of *Salmonella* and *Shigella* isolated from lettuce and green pepper

Isolates	No. of isolates	No. of resistant strain to*										
		Pen	Str	Pol B	Tet	Amo	Cef	Gen	Cip	Amp	Kan	Ch1
*Salmo*-	8	8	3	0	1	7	3	0	0	4	1	1
*Shigella*	24	22	8	6	10	19	10	1	1	18	1	2

* Pen, penicillin; Str, streptomycin; Pol B, polymyxin B; Tet, tetracycline; Amo, Amoxycillin; Cef, ceftriaxone; Gen, gentamycin, Cip, Ciproflaxin; Amp, ampicillin; Kan, kanamycin; Chl, chloramphenicol

All *Salmonella* and *Shigella* isolates showed multiple drug resistance (MDR) to three to seven more drugs. Five *Salmonella* isolates showed MDR against three drugs and seven *Shigella* isolates showed MDR to five drugs. The most dominant MDR pattern among both groups of isolates was Pen/Amo/Amp (Table 5).

**Table 5:** Multiple drug resistance of *Salmonella* and *Shigella* isolated from lettuce and green pepper

	Number of antibiotic resisted	Number of resistant isolates		Drugs resisted
*Salmonelle* *(*8 isolates)	Three	5	1	Pen/Str/Amo
			3	Pen/Amo/Amp
			1	Pen/Str/Cef
	Four	2	1	Pen/Str/Amo/Cef
			1	Pen/Amo/Amp/Kan
	Seven	1	1	Pen/Str/Tet/Amo/Cef/Amp/Chl
				
*Shigella* (24 isolates)	Three	6	5	Pen/Amo/Amp
			1	Pen/Tet/Amo
	Four	4	1	Pen/Tet/Amo/Amp
			1	Pen/Pol B/Amo/Chl
			1	Pen/Amo/Cef/Amp
			1	Pen/Cef/Amp/Chl
	Five	7	2	Pen/Pol B/Tet/Amo/Amp
			1	Pen/Str/Amo/Cef/Amp
			1	Pen/Pol B/Tet/Amo/Cef
			1	Pen/Str/Tet/Amo/Amp
			1	Pen/Amo/Cef/Amp/Chl
			1	Pen/Str/Amo/Cef/Gen
	Six	4	1	Pen/Str/Pol B/Amo/Cef/Amp
			1	Pen/Str/Amo/Cef/Amp/Chl
			1	Pen/Str/Tet/Amo/Cef/Amp
			1	Pen/Str/Pol B/Tet/Amo/Amp
	Seven	3	2	Pen/Str/Tet/Amo/Cef/Amp/Kan
			1	Pen/Str/Pol B/Amo/Cef/Cip/Amp

## Discussion

The majority of lettuce and green pepper samples considered in this study had high microbial load and, in some cases, even pathogens were isolated. As these are salad vegetables, they do not get any further heat treatment. The only control mechanisms are thorough washing and use of food grade chemicals to kill the microorganisms. Because of the high initial counts, reduction of number by washing or chemical treatment may still leave a good proportion of the microorganisms unaffected.

The mean aerobic mesophilic count observed in our samples were relatively higher than that for lettuce and lower for green pepper samples from Morocco (14). It was also also reported that aerobic mesophilic count between log 3 to log 8 cfu/g for lettuces to be served in Spain university restaurants (15). A higher mean aerobic mesophilic count of log 2 to log 10 was also reported from vegetable in another study (16).

The count of Enterobacteriaceae and coliforms, in particular, observed in this study was higher in our salad vegetable samples. Similar counts of Enterobacteriacea were reported in lettuce and green pepper from Morocco (14). Other reports showed lower coliform counts (15–16) in lettuce. The high level of enterobactericeae in our samples might indicate that the water used for irrigation could be heavily contaminated with faecal material from sewerage effluent.

Over 80% of the green pepper and lettuce samples harbored staphylococci with counts ranging from log 4 to log 6 cfu/g. The high level of contamination by *Staphylococcus* is a point of concern as possible presence of toxin producing Staphylococcus strains, if not controlled by other treatments, may cause staphylococcal food poisoning. It was reported that production of enterotoxin occurs at *Staphylococcus aureus* count of 10^6^ cfu/g (17). Staphylococci are common in products handled by hand (4), thus the high staphylococci load of lettuce observed in this study could be due to unhygienic handling.

The mean bacterial spore counts of lettuce in our study were ≤ log 2 cfu/g. Kelly and Kroll (18) reported a mean bacterial spore count of log 4.5 for enumeration of bacterial spores in food, which was markedly higher than the one obtained in our sample.

As green vegetables treated with food grade chemicals do not support the proliferation of spore forming bacteria (4), their presence at this level may not be considered hazardous.

High count of aerobic mesophilic bacteria, Enterobacteriaceae, coliforms, staphylococci, yeasts, molds and bacterial spores indicated that the salad vegetables might be contaminated with microorganism during growth, harvesting, or distribution. Thus, thorough cleaning and use of the right types and concentrations of food grade chemicals for cleaning or as salad dressings should be practiced to make the vegetables fit for consumption.

There was high variability in the counts of all microbial groups within the samples of each lettuce. This shows the lack of consistent washing and sanitation practices. In addition, there is an increased potential for vegetables to become contaminated with pathogenic species during production and processing as there is no system of microbiological control of the raw vegetable or the processed one.

*Shigella* was isolated from eight (12.5%) samples of lettuces and 16 (25%) samples of green peppers. *Shigella* was not detected in any of the lettuces served in Spain University restaurants (15). Onyemeluk and Njoku-obi (25) isolated *Shigella* from 5.6% of the samples in Nigeria. The detection of *Shigella* from lettuces revealed inadequacy concerning quality and safety of these products.

*Salmonella* spp. was isolated from a few samples of lettuce and green peppers. *Salmonella* was not detected in a range of commercially available organic vegetable samples taken form Northern Ireland (19) and from lettuces in Spain University restaurants (15). However, it was isolated from vegetable sample in Spain (20). Absence of *Salmonella* in organically grown vegetables indicated thorough cleaning using the appropriate chemicals.

The presence of *Salmonella* in 25 g of a sample examined is regarded as potentially hazardous to consumers, and is unacceptable for consumption (21). Rajkowski and Fan (22) also isolated *Salmonella* from imported and domestic lettuce samples and suggested that contamination with human pathogen could occur during the growth of the produce using bovine manure fertilizer, contaminated water or cross contamination during the cutting of the lettuce as the cut of lettuce can harbor and support the growth of food borne pathogen due to nutrients from leakage of plant cellular material.

The number of our *Salmonella* isolates was too small to make any valid comparisons. All isolates were resistant to Pen and most to Amo and Amp. Resistance to the other drugs used in this study was lower than that reported from Malaysia (23) and Brazil (24).

The finding of MDR *Salmonella* in our study was markedly higher than those isolate from Malaysia and Brazil (23–24) The investigation of multi-drug resistant *Salmonella* is relevant to gain an understanding of the epidemiology of emerging resistant *Salmonella* servovars.

Strains of *Shigella* isolates from fresh produce resistant to various commonly used antibiotics have been reported from various parts of the world (25). The resistance of our *Shigella* isolates to Str, Chl, Tet (34%) was lower than that reported from Nigeria (52%) but resistance to Amp was higher (25). Meanwhile, resistance to Tet was higher than that reported from Korea (26). Resistance of *Salmonella* and *Shigella* isolates to specific drugs could possibly be due to dissemination of drug resistance in the environment arising from the misuse of antibiotics among the general population.

Multi-drug resistant *Shigella* has been reported to be increasing in incidence worldwide (25). The frequency of MDR *Shigella* in our study was much higher than those isolates from Nigeria (25). It has been speculated that this may result from selection of resistant mutants through the indiscriminate and widespread use of antibiotics in the study area. The prevalence of drug resistance pattern in *Shigella* observed here and elsewhere indicates the futility of routine antibiotic therapy in bacillary dysentery.

This study showed that lettuce and green pepper are heavily contaminated with a variety of microbial groups and enteric pathogens were isolated from some samples. As their preparation in salads does not require further heat treatment, it is important to thoroughly wash such vegetables and dip them in food grade antibacterial chemicals for a good time to eliminate pathogens and significantly reduce the microbial load.
